# Monocyte-Derived Dendritic Cells Dictate the Memory Differentiation of CD8^+^ T Cells During Acute Infection

**DOI:** 10.3389/fimmu.2019.01887

**Published:** 2019-08-16

**Authors:** Kwang-Soo Shin, Insu Jeon, Byung-Seok Kim, Il-Kyu Kim, Young-Jun Park, Choong-Hyun Koh, Boyeong Song, Jeong-Mi Lee, Jiyoung Lim, Eun-Ah Bae, Hyungseok Seo, Young Ho Ban, Sang-Jun Ha, Chang-Yuil Kang

**Affiliations:** ^1^Laboratory of Immunology, College of Pharmacy, Seoul National University, Seoul, South Korea; ^2^Laboratory of Immunology, Department of Molecular Medicine and Biopharmaceutical Sciences, Graduate School of Convergence Science and Technology, Seoul National University, Seoul, South Korea; ^3^Laboratory of Immune Regulation, College of Pharmacy, Seoul National University, Seoul, South Korea; ^4^Department of Biochemistry, College of Life Science and Biotechnology, Yonsei University, Seoul, South Korea

**Keywords:** monocyte-derived dendritic cells, memory CD8^+^ T cells, IFN-γ, LCMV, acute infection

## Abstract

Monocyte-derived dendritic cells (moDCs) have been shown to robustly expand during infection; however, their roles in anti-infectious immunity remain unclear. Here, we found that moDCs were dramatically increased in the secondary lymphoid organs during acute LCMV infection in an interferon-γ (IFN-γ)-dependent manner. We also found that priming by moDCs enhanced the differentiation of memory CD8^+^ T cells compared to differentiation primed by conventional dendritic cells (cDCs) through upregulation of Eomesodermin (Eomes) and T cell factor-1 (TCF-1) expression in CD8^+^ T cells. Consequently, impaired memory formation of CD8^+^ T cells in mice that had reduced numbers of moDCs led to defective clearance of pathogens upon rechallenge. Mechanistically, attenuated interleukin-2 (IL-2) signaling in CD8^+^ T cells primed by moDCs was responsible for the enhanced memory programming of CD8^+^ T cells. Therefore, our findings unveil a specialization of the antigen-presenting cell subsets in the fate determination of CD8^+^ T cells during infection and pave the way for the development of a novel therapeutic intervention on infection.

## Introduction

CD8^+^ T cells play a dominant role in the elimination of infectious pathogens. Following their primary activation, CD8^+^ T cells undergo a fate determination between short-lived effector cells (SLECs) or memory-precursor effector cells (MPECs) (1). It has been suggested that various non-mutually exclusive factors direct the CD8^+^ T cell fate decision (2). For example, T cell receptor (TCR) signal quantity and quality determine the diversification of progeny from CD8^+^ T cells. A strong TCR signal licenses CD8^+^ T cells to undergo effector-prone differentiation, whereas a weak TCR signal diverts the fate of activated CD8^+^ T cells to memory-like cells (2, 3). Recent studies have proposed that these events result from complex transcriptional regulation (4). Indeed, T-bet and Blimp-1 have been shown to drive the fate of CD8^+^ T cells into effector cells, while Eomes and TCF1 are involved in differentiation into memory-like cells (1, 5–7). The TCR signal strength is governed by TCR-MHC interaction and co-stimulation delivered by various antigen-presenting cells (APCs) in the secondary lymphoid organs (SLOs); however, the contributions of each APC to the diversification of the CD8^+^ T cell fate remains poorly characterized.

Upon infection, myeloid cells robustly expand and are recruited to the inflamed sites to clear pathogens or infected cells (8, 9). Myeloid cells are crucial not only for innate immunity but also for initiation of adaptive immune responses. Among them, monocytes have been classically considered to be the major progenitors of macrophages that take part in the clearance of cellular debris and pathogens (10). However, it has recently become evident that adult tissue macrophages mainly develop from the yolk sac (or fetal liver)-derived progenitor cells during embryogenesis (11). Thus, the differentiation and function of monocytes during infection requires further investigation (10). During infection and inflammation, monocytes further differentiate into DC-like cells, which are referred to as monocyte-derived DCs (moDCs) or TNF- and iNOS-producing DCs (Tip-DCs) depending on the context (12, 13). Previous reports have proposed that common monocyte progenitor cells (cMoPs) in the bone marrow (BM) are progenitors of moDCs (14, 15). However, the specific mechanism that drives the differentiation of cMoPs into moDCs needs additional investigation.

Through rapid and robust myelopoiesis upon infection, monocytes and moDCs preferentially populate the antigen-presenting cell (APC) pool in the SLOs. Monocytes and moDCs are known to initiate CD8^+^ T cell responses by antigen presentation; their roles have been investigated and conflicting results were obtained in various animal models (16). For example, tumor-infiltrating moDCs have been shown to prime CD8^+^ T cells and induce anti-tumor immunity (15). In contrast, monocytic cells in chronic infections abrogate the induction of anti-infectious CD8^+^ T cell responses (17). Therefore, specific contribution of monocytes and moDCs to the differentiation of CD8^+^ T cells remains poorly understood.

In this report, we investigated the role of moDCs in CD8^+^ T cell fate determination during acute infection. We found that moDCs were expanded during *lymphocytic choriomeningitis virus* (LCMV) infection; in the case of bone marrow progenitor cells (BMPs), cMoPs differentiated into moDCs in an IFN-γ-dependent manner. In addition, CD8^+^ T cells that were primed in *Ccr2*^−/−^ mice, which have reduced numbers of moDCs in SLOs, could not properly develop into memory cells and underwent effector-prone differentiation during the expansion phase of infection. Moreover, *Ccr2*^−/−^ mice have defects in pathogen clearance upon reinfection due to the defective differentiation and survival of memory CD8^+^ T cells. Finally, attenuated IL-2 signals provided by moDCs were responsible for the enhanced memory differentiation of CD8^+^ T cells.

## Materials and Methods

### Animals

C57BL/6J mice were purchased from the Institute of Medical Science at the University of Tokyo and Balb/c mice from Charles River Laboratory. OT-I [C57BL/6-Tg(TcraTcrb)1100Mjb/J], P14 [B6;D2-Tg(TcrLCMV)327Sdz/JDvsJ], *Ccr2*^−/−^ (B6.129S4-Ccr2^tm1Ifc^/J), *Ifng*^−/−^ [C.129S7(B6)-Ifng^tm1Ts^/J], and CD45.1 (B6.SJL-Ptprc^a^Pepc^b^/BoyJ) mice were purchased from Jackson Laboratory. CD45.1^+^ P14 mice were obtained by crossbreeding CD45.1^+^ mice and C57BL/6J mice. Age (6 to 12 weeks) and sex-matched mice were used for all experiments. All mice were bred and maintained under specific pathogen-free conditions in the Animal Facility of Seoul National University. Experiments with infectious pathogen were performed in the ABL2 vivarium of Seoul National University. All animal protocols were approved by the Institutional Animal Care and Use Committee (IACUC) of Seoul National University.

### Infections and IFN-γ Neutralization

For primary infection, mice were injected intraperitoneally (i.p.) with LCMV-Arm [2 × 10^5^ plaque-forming units (PFU)] or intravenously (i.v.) with GP_33−41_-expressing *Listeria monocytogenes* [Lm-GP33, 5,000 colony-forming units (CFU)], which were generously donated from Yonsei University. To analyze the host protection capacity of memory cells, mice were infected with Lm-GP33 (5,000 CFU). To neutralize IFN-γ *in vivo*, mice were treated i.p. with 500 μg anti-IFN-γ mAb (HB170, ATCC) at day 1, 4, and 7 p.i., for LCMV-Arm infection, and at day −1 and 1 p.i., for Lm-GP33 infection. All experimental methods with infectious pathogens were reviewed and approved by the Institutional Biosafety Committee (IBC) of Seoul National University.

### Flow Cytometry

Spleen and peripheral lymph nodes were isolated from mice and homogenized using a 70 μm cell strainer (BD Biosciences). Bone marrow cells were isolated by flushing the tibia and femur of mice with a 1 ml syringe. PBMCs in the blood were isolated using Hispaque-1077 (Sigma-Aldrich) following the manufacturer's protocol. Red blood cells (RBC) of single cell suspensions were lysed using RBC Lysis Buffer (BioLegend).

Abs used for flow cytometry were as follows: anti-IA/IE (M5/114.15.2), anti-CD11b (M1/70), anti-CD11c (N418), anti-Ly6G (1A8), anti-Ly6C (HK1.4), anti-CCR2 (475301, R&D Systems), anti-CX3CR1 (SA011F11), anti-F4/80 (BM8, eBioscience), anti-CD64 (X54-5/7.1), anti-CD115 (AFS98), anti-CD135 (A2F10), anti-CD119 (2E2), anti-CD45.1 (A20), anti-CD45.2 (104), anti-H-2K^b^ (AF6-88.5), anti-H-D^b^ (KH95), anti-CD40 (3/23), anti-CD80 (16-10A1), anti-CD86 (GL-1), anti-PD-L1 (10F.9G2), anti-CD8α (53-6.7), anti-CD3ε (145-2C11), anti-CD25 (PC61), anti-CD62L (MEL-14), anti-KLRG1 (2F1/KLRG1), anti-CD127 (A7R34), anti-IFN-γ (XMG1.2), anti-TNF-α (MP6-XT22), anti-granzyme B (GB11), anti-Eomes (Dan11mag, eBioscience), anti-T-bet (4B10, eBioscience), and anti-TCF1 (C63D9, Cell Signaling). Abs were purchased from BioLegend unless otherwise described. Streptavidin-APC/Cy7 (BioLegend) and Alexa Fluor 647 goat anti-rabbit IgG (Invitrogen) were used for secondary staining.

Single cell suspensions were stained for surface molecules for 30 min at 4°C. Dead cells were excluded from analysis using Fixable Viability Dye (eBioscience). For intracellular cytokine staining, cells were restimulated with GP_33−41_ peptide (KAVYNFATC, 0.2 μg/ml, Genscript) in the presence of BD GolgiPlug (BD Biosciences) for 4 h, fixed and permeabilized using a Cytofix/Cytoperm kit (BD Biosciences) according to the manufacturer's instructions. To detect transcription factors, a fixation/permeabilization kit purchased from eBioscience was used. For analyzing cellular apoptosis, FITC Annexin V apoptosis Detection Kit I and Propidium Iodide Staining Solution (BD Biosciences) were used. Samples were collected using a FACS LSRFortessa X-20 or FACS Aria III (BD Biosciences), and data were analyzed with FlowJo software (Treestar).

### Cell Sorting

For sorting BMPs, BM cells of naïve mice were labeled with biotinylated anti-CD3ε, anti-CD19, anti-CD49b, and anti-Ly6G (all from Biolegend) followed by anti-biotin microbeads (Miltenyi Biotech) and isolated using a MACS LD column (Miltenyi Biotech). The collected cells were further sorted into each BMP subset: total BMPs (lin^−^c-kit^+^CD11b^−^Ly6C^−^), cMoPs (lin^−^c-kit^+^CD115^+^CD135^−^CD11b^−^Ly6C^+^), MDPs (lin^−^c-kit^+^CD115^+^CD135^+^CD11b^−^Ly6C^−^), CD135^+^ BMPs (lin^−^c-kit^+^CD115^−^CD135^+^CD11b^−^Ly6C^−^), and CD115^−^CD135^−^ BMPs (lin^−^c-kit^+^CD115^−^CD135^−^CD11b^−^Ly6C^−^).

To isolate cDCs and moDCs from infected mice, Lin^−^ cells of infected splenocytes (day 4 or 8 p.i.) were isolated using a MACS LD column and further sorted to each subset (cDCs as lin^−^IA/IE^+^CD11c^+^CD11b^−^CCR2^−^Ly6C^−^ and moDCs as lin^−^IA/IE^+^CD11c^−/int^CD11b^+^CCR2^+^Ly6C^+^).

CD45.1^+^ P14 cells used in the *in vitro* and *in vivo* experiments were enriched using anti-CD8a microbeads and a MACS LS column (Miltenyi Biotech) and further purified to CD45.1^+^CD8^+^ cells by cell sorting.

Cell sorting was conducted using a FACS Aria II or FACS Aria III. The purities of all sorted populations were >95%.

### ELISA

The IFN-γ concentration in mouse serum was measured using a mouse IFN-γ ELISA kit (BD Biosciences) according to the manufacturer's protocol. The IL-2 concentrations in the cocultures of T cells and APCs were measured using the following Abs: anti-IL-2 (JES6-1A12) for capture, biotinylated anti-IL-2 (JES6-5H4) and streptavidin-HRP for detection (all from BD Biosciences).

### BM Cell Differentiation Assay

To evaluate the differentiation patterns of BMPs *in vitro*, sorted total BMPs, cMoPs, MDPs, CD135^+^ BMPs, and CD115^−^CD135^−^ BMPs (1 × 10^4^ cells/well) were cultured for 4 to 6 days under specific conditions as follows: GM-CSF, GM-CSF plus IL-4, or GM-CSF plus IFN-γ (all from R&D systems). All recombinant cytokines were used at 20 ng/ml, and the culture medium was refreshed every 2 days.

To analyze the differentiation patterns of cMoPs *in vivo*, sorted cMoPs and non-cMoPs (2 × 10^5^ cells each/mouse) were adoptively transferred into LCMV-Arm-infected recipient mice at day 5 p.i., Donor cells were analyzed at day 3 post transfer (day 8 p.i.).

### *In vitro* APC:T Cell Coculture and *in vitro* Cytotoxicity Assay

The 1 × 10^4^ APCs (cDCs or moDCs) and 5 × 10^4^ P14 cells were cultured for 3 days in the presence of GP_33−41_ peptide. To determine the proliferation capacity of P14 cells, the cells were labeled with 5 μM of CellTrace Violet (CTV, Invitrogen) for 15 min prior to incubation. The cocultures in some experiments were treated with recombinant mouse IL-2 (10 ng/ml, Peprotech) or anti-IL-2 mAbs (10 μg/ml, JES6-1A12, eBioscience).

To measure the cytotoxicity of activated CD8^+^ T cells, equivalent numbers of purified live effector P14 cells from *in vitro* cocultures or infected mice were cocultured with ^51^Cr-labeled GP_33−41_-loaded EL4 cells (ATCC) for 4 h. Target cell specific lysis was measured by a Wallac 1470 Wizard automatic γ-counter (PerkinElmer) and calculated using the following equation; [(sample lysis count per minute (CPM)—spontaneous lysis CPM)/(Triton X-100-mediated lysis CPM—spontaneous lysis CPM)] × 100 (%).

### T Cell Adoptive Transfer

To examine the primary immune responses, 1 × 10^4^ purified CD8^+^ P14 cells from P14 splenocytes were adoptively transferred into WT or *Ccr2*^−/−^ mice and analyzed at the indicated time points. To establish the memory of P14 cells, 1 × 10^6^ P14 cells isolated from infected mice at day 8 p.i., were adoptively transferred to naïve recipient mice. To evaluate the memory-generation capacity of P14 cells primed by moDCs and cDCs *in vitro*, P14 cells were activated as indicated in the “*in vitro* APC:T cell coculture” section and then transferred to infected mice at day 8 p.i., (5 × 10^5^ cells/mouse).

### Quantitative Real-Time PCR

Total RNA of sorted P14 cells from infected mice at day 8 p.i., was isolated using TRIzol reagent and reverse-transcribed into cDNA using AmfiRivert II cDNA Synthesis Master Mix (Gendepot). Real-time PCR was performed with a SYBR Green real-time PCR kit (Takara) and LightCycler 1.5 instrument (Roche Diagnostics). Primers were purchased from Cosmo Genetech, and their sequences were as follows: mouse *Tbx21* (forward; 5′– ACA AGG GGG CTT CCA ACA AT −3′, reverse; 5′– TGC GTT CTG GTA GGC AGT CA −3′), mouse *Eomes* (forward; 5′– AGA ACC GTG CCA CAG ACC AA −3′, reverse; 5′– TCG TCA CAG GTT GCT GGA CA −3′), mouse *Tcf7* (forward; 5′– GCA CAC TTC GCA GAG ACT TT −3′, reverse; 5′– GTG GAC TGC TGA AAT GTT CG −3′), mouse *Prdm1* (forward; 5′– ACT CAG TCG CAT TTG ATG GC −3′, reverse; 5′– GGT CAG TAA GGC TCT TGG GT −3′), mouse *Il2* (forward; 5′– CAA CTG TGG TGG ACT TTC TG −3′, reverse; 5′– CCT TGG GGC TTA CAA AAA GAA −3′), and mouse *Hprt* (forward; 5′– AAG ACT TGC TCG AGA TGT CAT GAA −3′, reverse; 5′– ATC CAG CAG GTC AGC AAA GAA −3′). The value of each gene expression level was normalized to the expression level of mouse *Hprt*.

### Tissue Titration

Tissue titrations were conducted as described previously (18). Spleens of Lm-GP33 infected mice were prepared as a single-cell suspension and then treated with 1% Triton X-100 solution (Sigma-Aldrich). Each diluted suspension was plated on the BHI agar plates (BD Biosciences) and incubated overnight at 37°C. Colonies on the plates were counted next day, and the titers were calculated as CFUs per gram of spleen. Small fragments of the spleens from LCMV-Arm-infected mice were stored in DMEM with 1% FBS (Gibco) and were homogenized completely. Vero cells in 6-well plates were infected with each dilution of the spleen samples for 1 h, subsequently overlaid with Medium-199 (Gibco) agarose gel, and incubated for 4 days. Then, Vero cells were stained with Neutral red solution. Plaques were counted 2 days after the staining and the titers were calculated as PFUs per gram of spleen.

### Statistics

**Figure 1:**
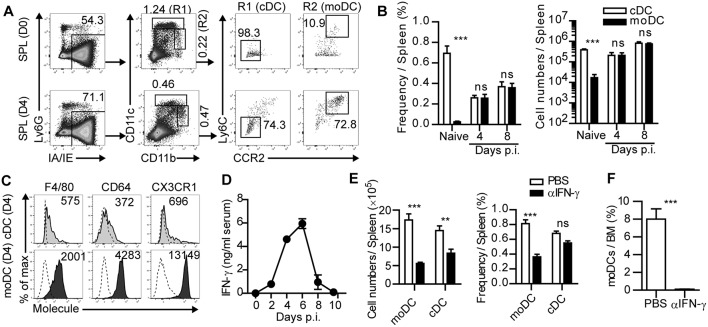
IFN-γ-dependent expansion of monocyte-derived dendritic cells during acute infection. **(A)** Gating strategies of cDCs and moDCs in the spleen of naïve or LCMV-Arm-infected mice. Numbers indicate the percentages within the gates. **(B)** Cell numbers and frequencies of cDCs and moDCs in the spleen during LCMV-Arm infection. **(C)** Expression patterns of indicated surface molecules on cDCs and moDCs in LCMV-Arm-infected mice at day 4 p.i., Numbers indicate the MFI values of each molecule. **(D)** Kinetics of IFN-γ levels in the serum of LCMV-Arm-infected mice. **(E,F)** LCMV-Arm-infected mice were treated with IFN-γ-neutralizing Ab. **(E)** Cell numbers (left) and frequencies (right) of cDCs and moDCs were measured in the indicated organs on day 8 p.i., and are shown as graph plots. **(F)** Frequency of moDCs in the BM. Data are representative of three independent experiments and are shown as the mean ± SEM. *n* = 5 per group at each time point. ^**^p < 0.01;^***^p < 0.001.

Statistical analysis of all data was conducted using GraphPad Prism 5 software (GraphPad Software, Inc.). The unpaired, two-tailed Student's *t*-test was used to compare two groups, and one-way ANOVA with Bonferroni's multiple comparisons was used to compare more than three groups. Two-way ANOVA with Bonferroni's multiple comparisons was used in Figure 1E, Supplementary Figures 1A,C. *P* < 0.05 were considered significant.

## Results

### IFN-γ-Dependent Expansion of Monocyte-Derived Dendritic Cells During Acute Infection

Various types of APCs in inflamed tissues and lymphoid organs are known to initiate adaptive immune responses during infection. Among those APCs, we sought to determine the role of moDCs in anti-infectious immune responses. Initially, we investigated the frequencies and numbers of moDCs and cDCs in lymphoid organs after acute LCMV (LCMV-Arm) infection. Based on a previous report (19), cDCs and moDCs in the spleen were defined by their cell surface marker expression patterns including Ly6G^−^IA/IE^+^CD11c^hi^CD11b^low/hi^Ly6C^−^CCR2^−^ cells and Ly6G^−^IA/IE^+^CD11c^low/int^CD11b^hi^Ly6C^+^CCR2^+^ cells, respectively (Figure 1A). moDCs were sparsely present in uninfected mice, while cDCs represented a dominant APC subset at steady state. However, moDCs rapidly accumulated in the spleen after LCMV-Arm infection and became an abundant APC population during the expansion phase of infection (Figures 1A,B). In addition, high expression levels of F4/80, CD64, and CX3CR1 in moDCs compared to those in cDCs indicated that moDCs were distinct from cDCs (Figure 1C).

We further investigated the underlying mechanism of the accumulation of moDCs during infection. Recent reports have demonstrated that IFN-γ induces the differentiation, migration and expansion of inflammatory monocyte lineage cells during infection (20–22). In this regard, the level of serum IFN-γ was elevated in LCMV-Arm-infected mice ~2 days prior to the accumulation of moDCs (Figure 1D). To determine whether the accumulation of moDCs was regulated by IFN-γ, we treated infected mice with IFN-γ-neutralizing Ab (Supplementary Figure 1A). IFN-γ neutralization led the slight increase in the viral loads in mice (Supplementary Figure 1B) (23). Although the absolute numbers of cDCs and moDCs were decreased along with reductions in the numbers of splenocytes, we found that the frequency of moDCs was markedly reduced in the spleen of mice that received IFN-γ-neutralizing Ab, while the frequency of cDCs did not decrease significantly (Figure 1E and Supplementary Figure 1C). Importantly, moDCs were almost completely absent in the BM of LCMV-Arm-infected mice with neutralized IFN-γ (Figure 1F). IFN-γ neutralization did not lead to the reductions of BMPs, but it rather induced the enhanced BMP frequencies (Supplementary Figure 1D). Furthermore, IFN-γ neutralization did not induce apoptotic death of moDCs (Supplementary Figure 1E). Thus, we concluded that the IFN-γ-dependent accumulation of moDCs in the periphery is due to the increased generation of the cells rather than the enhanced migration from the BM, their enhanced survival, or the effects of IFN-γ on the frequencies of BMPs. We also confirmed that IFN-γ is required for moDC generation in the spleen and BM during LCMV-Arm infection using IFN-γ-deficient mice (Supplementary Figure 2A). Additionally, we found that the frequency of moDCs was also increased in GP_33−41_-expressing *Listeria monocytogenes* (Lm-GP33)-infected mice (Supplementary Figures 2B,C). Consistent with the result on LCMV-Arm infection, accumulation of moDCs was dependent on IFN-γ during Lm-GP33 infection (Supplementary Figures 2B,C). Altogether, these results indicate that moDCs robustly expand in an IFN-γ-dependent manner during an acute viral and bacterial infection.

### IFN-γ Acts Directly on Common Monocyte Progenitor Cells and Promotes the Differentiation of moDCs

**Figure 2:**
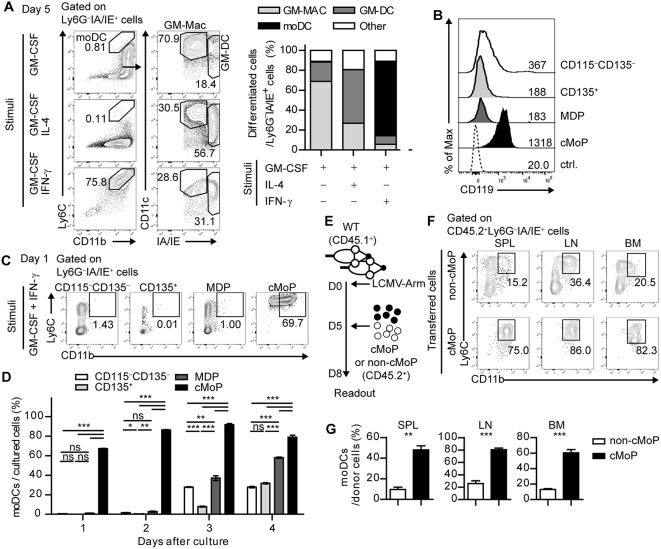
IFN-γ acts directly on common monocyte progenitor cells and promotes the differentiation of moDCs. **(A)** Differentiation patterns of total BMPs at day 5 under different stimuli (GM-CSF alone, GM-CSF + IL-4, and GM-CSF + IFN-γ are shown as flow cytometry plots (left) and graphs (right). Numbers in the plots indicate the percentages within the gates. **(B)** IFN-γR (CD119) expression levels of the BMP subsets. Numbers indicate the MFI values of each subset. **(C,D)** Daily differentiation of the subdivided BMP subsets into moDCs under GM-CSF and IFN-γ stimulation. **(C)** Representative flow cytometry plots of sorted BMP subsets after a day of culture **(D)** Graph shows the proportions of cells that differentiate into moDCs in each BMP subset. **(E–G)** Sorted cMoPs or non-cMoPs were transferred into LCMV-Arm-infected mice on day 5 p.i., and their differentiation into moDCs was analyzed 3 days later. **(E)** Experimental schedule. **(F–G)** Differentiation patterns of donor cells into moDCs in the indicated organs are shown as flow cytometry plots **(F)** and graphs **(G)**. Numbers in the plots indicate the percentages within the gates. Data are representative of two or three independent experiments and are shown as the mean ± SEM. ^*^p < 0.05; ^**^p < 0.01; ^***^p < 0.001.

The observed role of IFN-γ in the surge of moDCs in the periphery after acute infection prompted us to investigate whether IFN-γ can influence moDC generation from specific BMPs. To achieve this goal, we sorted lineage^−^c-kit^+^ BMPs from naïve mice and differentiated them with GM-CSF, GM-CSF plus IL-4, or GM-CSF plus IFN-γ. In line with a previous report, BMPs cultured with GM-CSF differentiated into two distinct populations including GM-Macs (CD11c^+^IA/IE^int^) and GM-DCs (CD11c^+^IA/IE^hi^), which represent monocyte-derived macrophages and dendritic cells, respectively (24). While the addition of IL-4 favors differentiation of GM-DCs, IFN-γ promoted the differentiation of Ly6C^+^CD11b^+^ cells instead of GM-Macs or GM-DCs (Figure 2A). Collectively, these results demonstrate that IFN-γ diverts the fate of BMPs from GM-Macs or GM-DCs to moDCs.

Next, we examined the subpopulation of BMPs that directly responds to IFN-γ. BMPs can be subdivided into four subpopulations based on cell surface expression of CD115 (M-CSFR) and CD135 (Flt3): CD115^−^CD135^+^ cells as CD135^+^ BMPs, CD115^+^CD135^+^ cells as monocyte-DC progenitors (MDPs), CD115^+^CD135^−^ cells as cMoPs, and CD115^−^CD135^−^ cells as resting CD115^−^CD135^−^ BMPs. Each BMP subpopulation expressed different levels of Ly6C and CD11b (Supplementary Figure 3A). Interestingly, cMoPs displayed a high expression level of CD119 (IFN-γR) compared with that in the other populations (Figure 2B). We isolated these cells and differentiated each subpopulation in the presence of GM-CSF and IFN-γ. In line with the high IFN-γR expression, only cMoPs immediately differentiated into CD11b^+^IA/IE^+^Ly6C^+^Ly6G^−^ moDC phenotype cells in response to IFN-γ in agreement with the earlier findings that suggested that cMoPs is a progenitor population of moDCs (Figures 2C,D and Supplementary Figure 3B) (14, 15). The differentiations of other populations into moDCs beginning on day 3 of the culture were likely due to a developmental hierarchy of BMPs. To directly investigate whether cMoPs can differentiate into moDCs, we sorted cMoPs and other resting BMPs (non-cMoPs) from naive mice and transferred them into LCMV-Arm-infected congenically marked (CD45.1^+^) recipient mice on day 5 post infection (p.i.) when IFN-γ was abundant. The frequencies of moDCs among donor cells were analyzed 3 days later (Figure 2E). Consistent with the *in vitro* experiments, the majority of cMoPs differentiated into moDCs, while non-cMoPs showed limited differentiation into moDCs (Figures 2F,G). Taken together, these data suggest that IFN-γ acts directly on cMoPs to promote their differentiation into moDCs in LCMV Arm-infected mice. The accumulation of cMoPs in mice that received IFN-γ-neutralizing Ab (Supplementary Figure 1B) could be explained by these results; cMoPs rarely received IFN-γ signaling, could not develop into moDCs, and consequently accumulated in the BM.

### CD8^+^ T Cells Primed by moDCs Have Reduced Effector Function Than Those Primed by cDCs

**Figure 3:**
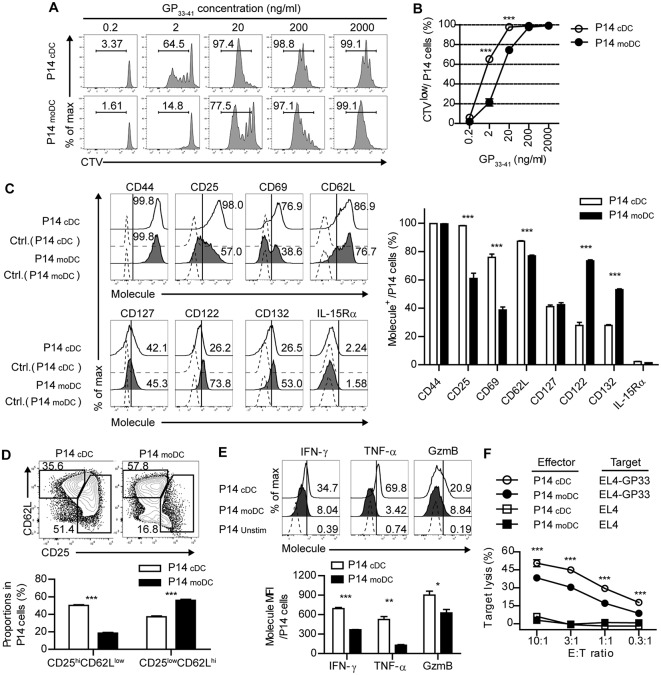
CD8^+^ T cells primed by moDCs have reduced effector function than those primed by cDCs. **(A,B)** Representative histograms **(A)** and graph **(B)** of CTV dilutions in P14 cells primed by cDCs or moDCs in the presence of different doses of GP_33−41_ peptide for 3 days. Numbers in the histograms indicate the percentage of cells that were divided at least once. **(C–F)** P14 cells were activated by cDCs or moDCs in the presence of 0.2 μg/ml GP_33−41_ peptide for 3 days. **(C)** Expression levels of indicated surface molecules are shown as histograms (right) and a graph plot (left). Numbers in the histograms indicate the percentages of positive cells for each molecule. **(D)** Coexpression of CD25 and CD62L on P14 cells primed by cDCs or moDCs are shown as flow cytometry plots (upper) and graph (lower). Numbers in the plots indicate the percentages within each gate. **(E)** Secretion levels of the indicated effector molecules in P14 cells that were primed by cDCs or moDCs are shown as histograms (upper) and graph (lower). Numbers in the histograms indicate the percentages of positive cells for each molecule. **(F)** *in vitro* target killing ability of P14 cells primed by cDCs or moDCs. Cr^51^-labeled GP_33−41_-loaded EL4 tumor cells were used as the target cells. Data are representative of three independent experiments and are shown as the mean ± SEM. ^*^p < 0.05;^**^p < 0.01; ^***^p < 0.001.

Given that moDCs become the dominant population of APCs during the expansion phase of acute viral infection, moDCs may be involved in modulating antiviral T cell responses. Thus, we determined whether infection-induced moDCs play an essential role in the virus-specific CD8^+^ T cell responses. To compare the ability of each DC subset to stimulate CD8^+^ T cell responses, we isolated moDCs and cDCs from the LCMV-Arm-infected mice on day 4 p.i., and cocultured the cells in the presence of various doses of cognate peptide (GP_33−41_) with Cell trace violet (CTV)-labeled P14 cells, which express TCRs that recognize the epitope peptide of the LCMV glycoprotein. moDCs were shown to have lower priming capacity than that of cDCs under low antigenic peptide stimulation (20 ng/ml), suggesting that moDCs deliver weak signals to CD8^+^ T cells (Figures 3A,B). Then, we compared the surface phenotypes of the P14 cells that were primed by each DC subset. Analysis of surface marker expression levels revealed that, while CD44 expressions were comparable between the two cell types, cDC-stimulated P14 cells (P14_cDC_) were more activated than moDC-stimulated P14 cells (P14_moDC_) as shown by elevated CD25 (IL-2Rα) and CD69 levels. Instead, P14_moDC_ showed upregulated expressions of CD122 (IL-2/15 Rβ) and CD132 (common γ chain) compared to P14_cDC._CD127 (IL-7R) levels were similar between P14_cDC_ and P14_moDC_, and IL-15Rα was rarely detectable in both cell types (Figure 3C). Interestingly, when P14 cells were plotted by their coexpressions of CD25 and CD62L, P14_moDC_ displayed a predominantly memory-like phenotype (CD25^low^CD62L^hi^), whereas P14_cDC_ had a higher fraction of effector-like cells (CD25^hi^CD62L^low^) (Figure 3D) (25, 26). Moreover, P14_moDC_ showed reduced expressions of effector molecules [IFN-γ, TNF-α, and granzyme B (GzmB)] compared with those of P14_cDC_ (Figure 3E). Similar results were obtained in the experiments using APCs isolated from infected mice on day 8 p.i., Day 8 moDCs were indistinguishable from day 4 moDCs in terms of T cell-stimulating capacity (Supplementary Figures 4A–D).

To directly compare antigen-specific cytolytic function of P14_moDC_ with that of P14_cDC_, we cocultured each type of CD8^+^ effector T cells with GP_33−41_-loaded EL4 tumor cells. Consistent with the expression levels of effector molecules, P14_moDC_ exhibited lower target killing ability than that of P14_cDC_ (Figure 3F). These data suggest that moDCs were not efficient in generation of effector CD8^+^ T cells but had an ability to induce memory precursor CD8^+^ T cells.

### Stimulation by moDCs Dictates the Developmental Program of Memory CD8^+^ T Cells by Transcriptional Regulation

**Figure 4:**
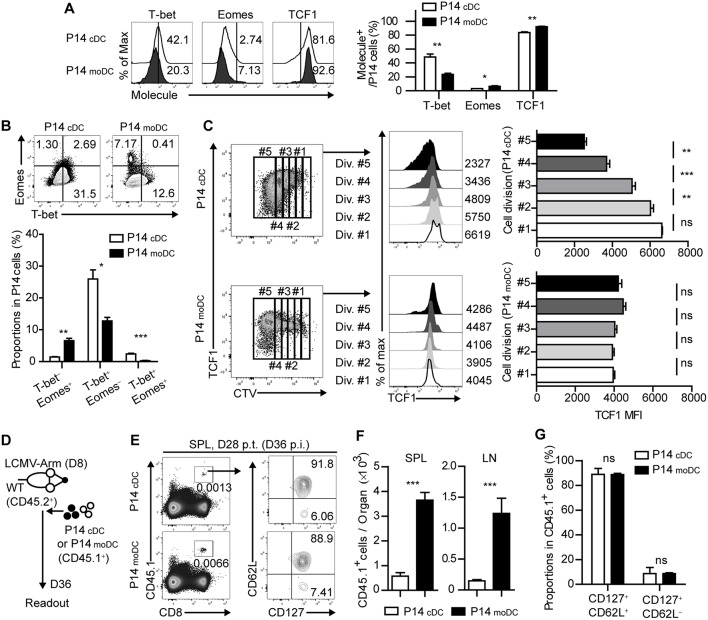
Stimulation by moDCs dictates the developmental program of memory CD8^+^ T cells by transcriptional regulation. **(A)** Expression levels of the indicated transcription factors in P14 cells primed by cDCs or moDCs are shown as histograms (upper) and graph (lower). Numbers in the histograms indicate the percentages of positive cells for each molecule. **(B)** Coexpressions of T-bet and Eomes in P14 cells primed by cDCs or moDCs are shown as flow cytometry plots (upper) and graph (lower). Numbers in the plots indicate the percentages of the cells in each quadrant. **(C)** P14 cells primed by cDCs or moDCs were gated by their cell division (left). TCF1 expression levels of each gate are shown as histograms (center) and graphs (right). Numbers in the histograms indicate the MFI values of TCF1 expression in each gate. **(D–G)** CD45.1^+^ P14 cells were primed *in vitro* by cDCs or moDCs, transferred to infected recipient mice on day 8 p.i., and analyzed on day 28 post transfer. **(D)** Experimental schedule. **(E)** Representative flow cytometry plots of donor cells in the spleens of recipient mice. **(F)** Graphs show the number of donor P14 cells in the indicated organs. **(G)** Graphs show the coexpressions of CD127 and CD62L of the donor cells in the spleen of the recipient mice. Data are representative of two or three independent experiments and are shown as the mean ± SEM. ^*^p < 0.05; ^**^p < 0.01; ^***^p < 0.001.

It has been shown that the fate determination of CD8^+^ T cells is regulated by the expression of several transcription factors (4). To further identify whether the signal delivered by moDCs directs CD8^+^ T cells to memory cells, we analyzed the transcriptional changes of P14_cDC_ and P14_moDC_. Interestingly, P14_moDC_ expressed low levels of T-bet and partly differentiated into Eomes^+^ cells while P14_cDC_ differentiated into T-bet^+^ effector cells (Figures 4A,B). Moreover, TCF1, a transcription factor associated with memory T cell differentiation (7), was maintained at relatively higher levels in P14_moDC_ compared to that in P14_cDC_ (Figure 4A). The TCF1 expression level in P14_moDC_ remained constant regardless of the number of cell divisions, whereas that in P14_cDC_ was inversely correlated with the number of cell divisions (Figure 4C). To evaluate the memory formation ability of CD8^+^ T cells activated by moDCs, equivalent numbers of the viable P14_cDC_ or P14_moDC_ were transferred into LCMV-infected recipient mice on day 8 p.i., and analyzed on day 28 post transfer (day 36 p.i.) (Figure 4D). The memory phenotype of P14_cDC_ and P14_moDC_ showed no difference at the memory time point; however, recipient mice that received P14_moDC_ had a higher number of donor cells than recipient mice that received P14_cDC_, suggesting that moDCs induced long-term survival of P14_moDC_ (Figures 4E–G). Overall, these results suggest that moDC stimulation diverts the fate of CD8^+^ T cells to memory cells.

### CD8^+^ T Cells Fail to Differentiate Into MPECs in CCR2-Deficient Mice

**Figure 5:**
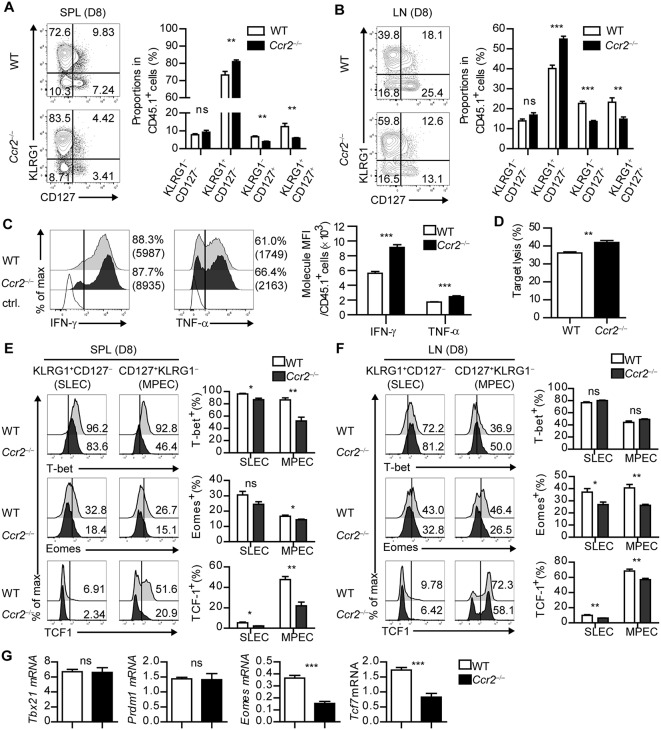
CD8^+^ T cells fail to differentiate into MPECs in CCR2-deficient mice. **(A,B)** Coexpressions of KLRG1 and CD127 of CD45.1^+^ P14 cells in the spleen and LN of LCMV-Arm-infected WT and *Ccr2*^−/−^ mice on day 8 p.i., are shown as flow cytometry plots (left) and graph (right). Numbers in the plots indicate the percentage of cells within each quadrant. **(C)** Secretion levels of IFN-γ and TNF-α in CD45.1^+^ P14 cells in the spleen of WT and *Ccr2*^−/−^ mice on day 8 p.i., are shown as histograms (left) and graph (right). **(D)** *In vitro* target killing ability of CD45.1^+^ P14 cells from WT and *Ccr2*^−/−^ splenocytes on day 8 p.i., Cr^51^-labeled GP_33−41_-loaded EL4 tumor cells were used as the target cells. **(E,F)** Expression levels of T-bet, Eomes and TCF1 in SLECs and MPECs of CD45.1^+^ P14 cells from WT and *Ccr2*^−/−^ splenocytes and LN cells on day 8 p.i., are shown as histograms (upper) and graph (lower). Numbers in the histograms indicate the percentage of positive cells for each molecule. **(G)** Gene expression levels of *Tbx21, Prdm1, Eomes*, and *Tcf7* in CD45.1^+^ P14 cells from WT and *Ccr2*^−/−^ splenocytes on day 8 p.i., were measured by real-time PCR. Expression levels were normalized to m*Hprt*. Data are representative of three independent experiments and are shown as the mean ± SEM. *n* = 5 per group. ^*^p < 0.05; ^**^p < 0.01; ^***^p < 0.001.

To determine the role of moDCs in the fate determination of CD8^+^ T cells during acute viral infection, we used CCR2-deficient (*Ccr2*^−/−^) mice. While the frequency and composition of cDCs was comparable to that in the WT mice, moDCs in SLOs were dramatically reduced in the *Ccr2*^−/−^ mice compared to WT mice during infection (Supplementary Figures 5A,B). The virus titers of *Ccr2*^−/−^ mice and WT mice showed no differences during the acute phase of infection (Supplementary Figure 5C). We transferred CD45.1^+^ P14 cells into WT and *Ccr2*^−/−^ mice, which were subsequently infected with LCMV-Arm, and analyzed the transferred P14 cells on day 8 p.i., To dissect the fate of CD8^+^ T cells, we subdivided the donor T cells by the distinct expression pattern of KLRG1 and CD127. When WT and *Ccr2*^−/−^ mice bearing CD45.1^+^ P14 cells were infected, the frequency of KLRG1^+^CD127^−^ cells, identified as short-lived effector cells (SLECs), was significantly increased in P14 cells in *Ccr2*^−/−^ mice compared with that in WT mice. In addition, the frequency of KLRG1^−^CD127^+^ cells, which represent memory precursor cells (MPECs), was significantly reduced in *Ccr2*^−/−^ hosts, suggesting that CCR2^+^ cells contributed to the efficient generation of memory CD8^+^ T cells during infection (Figures 5A,B). We also examined the effector function of P14 cells in WT and *Ccr2*^−/−^ mice. The intensity, but not the frequency, of IFN-γ or TNF-α-producing T cells among P14 cells was elevated in *Ccr2*^−/−^ mice compared with that in WT mice (Figure 5C). Furthermore, P14 cells from *Ccr2*^−/−^ mice showed more potent target cell killing activity than those from WT mice (Figure 5D).

To determine whether the altered effector/memory differentiation of virus-specific CD8^+^ T cells in *Ccr2*^−/−^ mice reflects molecular changes in CD8^+^ T cells, we compared the transcriptional profile of SLECs and MPECs in transferred P14 cells of WT and *Ccr2*^−/−^ mice. Consistent with the results of the *in vitro* studies (Figure 4), the expressions of Eomes and TCF1 in each P14 cell subset were decreased in *Ccr2*^−/−^ mice compared with those in WT mice and the effect was more prominent in MPECs (Figures 5E,F). Despite the reduced numbers of moDCs, SLECs, and MPECs of P14 cells in *Ccr2*^−/−^ mice had low levels of T-bet expression, suggesting that other factors could have contributed to the transcriptional changes in CD8^+^ T cells. In addition, the expressions of *Tcf7* and *Eomes* in P14 cells were significantly reduced in *Ccr2*^−/−^ recipient mice compared to WT mice, while the expressions of *Tbx21* and *Prdm1* were similar (Figure 5G). Collectively, these results suggest that moDCs can induce memory precursor cells from naïve CD8^+^ T cells by regulation of transcription factor expression including Eomes and TCF1.

### CD8^+^ T Cells Primed in CCR2-Deficient Mice Cannot Respond to Reinfection

Then, we analyzed transferred P14 cells at the memory phase of infection. On day 35 p.i., P14 cells were barely detectable in the spleen and LN of *Ccr2*^−/−^ mice (Supplementary Figures 6A,C), which may be due to reduced frequencies of MPECs in *Ccr2*^−/−^ mice at the early phase of infection. However, the phenotype of the remaining P14 cells in WT and *Ccr2*^−/−^ mice was similar except that fraction of CD127^−^CD62L^−^ cells was slightly increased in splenic P14 cells of *Ccr2*^−/−^ mice (Supplementary Figures 6B,D). Additionally, P14 memory cells in *Ccr2*^−/−^ mice had similar inflammatory potency compared with those in WT mice (Supplementary Figure 6E). These results suggest that moDCs play a major role in maintaining the memory CD8^+^ T cell pool without affecting their functions.

**Figure 6:**
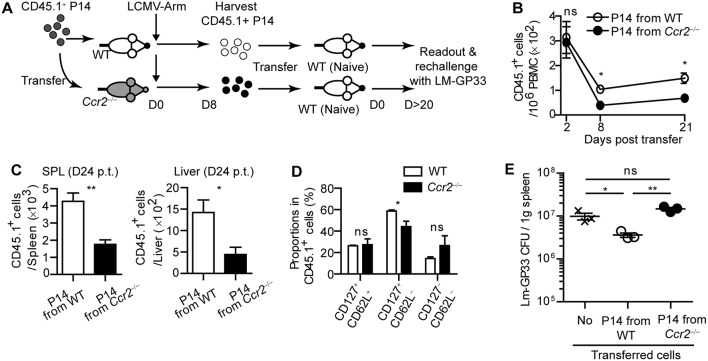
CD8^+^ T cells primed in CCR2-deficient mice cannot respond to reinfection. Effector P14 cells of WT and *Ccr2*^−/−^ mice at day 8 p.i., were sorted and equivalent numbers of the cells were transferred into naïve mice. Recipient mice were analyzed at least 20 days after transfer. **(A)** Experimental schedule. **(B)** Temporally enumerated CD45.1^+^ P14 cells in blood PBMCs of recipient mice after the transfer of effector P14 cells from WT and *Ccr2*^−/−^ mice. **(C)** The frequencies of CD45.1^+^ P14 cells in the spleen (left) and liver (right) of recipient mice on day 24 post transfer of P14 cells from WT and *Ccr2*^−/−^ mice. **(D)** The memory phenotypes of CD45.1^+^ P14 cells in the spleen of recipient mice on day 24 post transfer of P14 cells from WT and *Ccr2*^−/−^ mice. **(E)** Recipient mice were challenged with Lm-GP33 at day 26 post transfer. Graph shows the bacterial titers in the spleen of recipient mice at day 3 after rechallenge. Data are representative of two independent experiments and are shown as the mean ± SEM. *n* = 3–4 per group. ^*^p < 0.05; ^**^p < 0.01.

To examine whether virus-specific CD8^+^ T cell priming by moDCs leads to enhanced protection against rechallenge, we harvested CD45.1^+^ effector P14 cells from WT or *Ccr2*^−/−^ mice on day 8 post LCMV-Arm infection. Then, we transferred an equivalent number of each type of P14 cells into naive WT recipient mice to establish LCMV-specific memory CD8^+^ T cells in the hosts (Figure 6A). As expected, P14 cells primed in WT mice preferentially survived in the hosts compared to those primed in *Ccr2*^−/−^ mice, as shown by reduced numbers of CD45.1^+^ P14 cells from *Ccr2*^−/−^ mice in the blood, spleen, and liver in recipient mice over 20 days post transfer, with a minor alteration in the phenotype of these cells (Figures 6B–D). We infected recipient mice with Lm-GP33 on day 26 after transfer. Consistent with the reduced memory P14 cell numbers before reinfection, mice that received effector P14 cells from *Ccr2*^−/−^ mice could not control bacterial burden while mice that received P14 cells from WT mice showed reduced bacterial burden compared with the untransferred control (Figure 6E). Taken together, our data suggest that moDCs induced by infection are crucial in the long-term survival of CD8^+^ T cells, thus enabling efficient clearance of pathogens by the hosts upon reinfection.

### Defective IL-2 Signaling Grants moDCs an Ability to Induce Memory CD8^+^ T Cells

**Figure 7:**
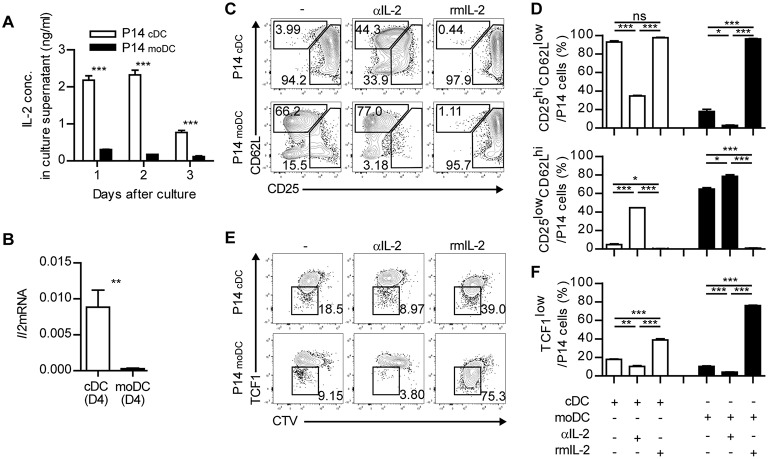
Defective IL-2 signaling grants moDCs an ability to induce memory CD8^+^ T cells. **(A)** IL-2 concentrations in the supernatant from the cultures of P14 cells with cDCs or moDCs. **(B)** Gene expression levels of *Il2* in cDCs and moDCs isolated from LCMV-Arm-infected splenocytes. **(C–F)** Recombinant IL-2 or anti-IL-2 mAbs were added to the cultures of P14 cells with moDCs or cDCs. **(C,D)** Coexpressions of CD25 and CD62L under each condition are shown as flow cytometry plots **(C)** and graphs **(D)**. **(E,F)** Expression levels of TCF1 are shown as flow cytometry plots **(E)** and graph **(F)**. Numbers in the plots indicate the percentage within each gate. Data are representative of two independent experiments and are shown as the mean ± SEM. ^*^p < 0.05; ^**^p < 0.01; ^***^p < 0.001.

Finally, we sought to investigate the underlying mechanisms that mediate the differentiation of memory-precursor cells during the interaction between CD8^+^ T cells and moDCs. Three signals have been known to be responsible for the initial activation of CD8^+^ T cells: TCR-MHC interaction, co-stimulation and cytokine signaling (27, 28). moDCs showed no defect in the expression of MHCI and co-stimulatory molecules (CD40, CD80, and CD86) compared to those of cDCs (Supplementary Figure 7). Thus, we hypothesized that defective cytokine signaling caused moDCs to deliver relatively weak signals to CD8^+^ T cells. Interestingly, the differentiation patterns of CD8^+^ T cells primed in the presence of a high dose of IL-2 and in the presence of a low dose of IL-2 resembled P14_cDC_ and P14_moDC_, respectively (29, 30). Accordingly, the levels of IL-2 in the supernatants of P14 cells cocultured with cDCs were higher than in those cocultured with moDCs (Figure 7A). Moreover, only cDCs expressed IL-2 at the transcript level (Figure 7B). In addition, an analysis of IL-2 secretion levels in the cocultures 12 h after culture revealed that cDCs produced more IL-2 than moDCs and P14_cDC_ expressed high levels of IL-2 compared to P14_moDC_ (Supplementary Figure 8). Therefore, we hypothesized that defective IL-2 signaling is responsible for the differentiation of CD8^+^ T cells into CD25^low^CD62L^hi^ memory precursor cells.

To test this possibility, we cultured CD8^+^ T cells with cDCs or moDCs in the presence of anti-IL-2 mAbs (αIL-2) or recombinant IL-2 (rmIL-2). In agreement with our hypothesis, P14_cDC_ cells cultured with αIL-2 predominantly differentiated into CD25^low^CD62L^hi^ memory phenotype cells suggesting that IL-2 was required for the full effector cell differentiation of P14 cells (Figures 7C,D). Due to the endogenous IL-2 secretion of P14_cDC_, adding rmIL-2 to cocultures showed no effect on the surface phenotypes of P14_cDC_. P14_moDC_, which were mainly differentiated into CD25^low^CD62L^hi^ memory precursor cells, were converted into CD25^hi^CD62L^low^ effector cells by adding rmIL-2 to the cocultures. We also found that the culture of P14_cDC_ with αIL-2 abrogated the downregulation of TCF1, whereas the culture of P14_moDC_ with rmIL-2 showed dramatic suppression of TCF1 expression (Figures 7E,F). Collectively, these results suggest that IL-2 signaling is important for modulation of the fate of CD8^+^ T cells during priming and that moDCs promote memory-prone differentiation of CD8^+^ T cells by delivering defective signaling such as IL-2.

## Discussion

Understanding the mechanism of the influence of myeloid cells on the development of T cell immunity during infection is important for establishing strategies for development of vaccines against infections. Here, we demonstrate that moDCs dramatically expand in an IFN-γ dependent manner during acute infection. More importantly, CD8^+^ T cells activated by moDCs preferentially differentiate to memory cells by inducing Eomes expression and maintaining TCF1 expression, while those cells that are primed by cDCs undergo effector-prone differentiation through upregulation of T-bet and downregulation of TCF1. Moreover, we found that the difference in IL-2 production between the two APC subsets results in the different outcomes of the primed CD8^+^ T cells.

APC subsets with distinct properties have been proposed to be crucial for the differentiation of T cells (31, 32). Our study suggests that the interaction between T cells and distinct APC subsets during priming regulates the fate of CD8^+^ T cells. Since cDCs are enriched in the SLOs during the initial phase of acute viral infection, they may serve as the primary professional APCs that prime CD8^+^ T cells resulting in the predominant generation of effector T cells. As the increased effector CD8^+^ T cell populations primed by cDCs clear the pathogen by producing effector molecules including IFN-γ, the elevated IFN-γ also contributes to the accumulation of moDCs in the SLOs. As a result, moDCs become abundant in the SLOs during the expansion phases of infection. It has been suggested that decreased TCR stimulation strength due to the reduced antigen burden after pathogen clearance results in the differentiation of memory CD8^+^ T cells (2, 4); however, our data suggest another possibility that changes in the APC subset composition also contribute to the effector/memory fate determination of CD8^+^ T cells. Our data also revealed that, in addition to its direct effect on effector CD8^+^ T cell expansion (33), IFN-γ can also influence memory CD8^+^ T cell generation indirectly by inducing moDCs. In line with this notion, generation of memory CD8^+^ T cell was abrogated in IFN-γR-deficient mice (34, 35). Therefore, we suggest that the changes in APC composition as well as the environment, including antigen abundance and CD4 T cell help, contribute to memory CD8^+^ T cell formation (36).

Previous studies have shown that IFN-γ induces the differentiation of moDCs by influencing cMoPs (14, 15, 37). We confirmed this finding and found that IFN-γ potentiated moDC differentiation by directly acting on cMoPs. Although granulocyte-macrophage progenitor cells (GMPs) are known to express IFN-γR (38), we extended this finding by showing that IFN-γR^hi^ cMoPs were the major precursor for moDC. A previous study has demonstrated that IFN-γ provokes the expansion of myeloid cells through an indirect pathway (39). We found an additional pathway by which IFN-γ induces the expansion of monocyte-derived cells, such as moDCs, via direct action on cMoPs. It is tempting to investigate why and how cMoPs selectively upregulate CD119 expression during monopoiesis.

Among the different signals that regulate the fate of CD8^+^ T cells, defective IL-2 signaling in the cocultures of moDCs and CD8^+^ T cells was shown to lead the memory-prone differentiation of CD8^+^ T cells. It has been suggested that the fates of CD8^+^ T cells are regulated by the differential exposures to IL-2 signals (40). Indeed, IL-2 signaling has been known to induce the terminal differentiation of CD8^+^ T cells by activating STAT-5 and consequently abrogate the memory CD8^+^ T cell generation (41). IL-2 intracellular cytokine staining showed that P14 T cells expressed higher levels of IL-2 than DCs in the cocultures, suggesting that CD8^+^ T cells were the main source of IL-2 in the cocultures (Supplementary Figure 8). In addition, we found that P14_cDC_ secreted more IL-2 than P14_moDC_. It leads to the question of why CD8^+^ T cells primed by cDCs secrete higher levels of IL-2 than those primed by moDCs. Interestingly, previous studies reported that the IL-2 production by DCs allows them to stimulate T cells. DC-derived IL-2 accumulation at the DC-T contact site has been considered to be important in the stimulation of T cells (42–44). From the viewpoint of these findings, our data suggest that cDCs, potent IL-2 providers, induce robust IL-2 production from CD8^+^ T cells and dictate terminal differentiation of CD8^+^ T cells. On the other hand, moDCs stimulate CD8^+^ T cells suboptimally via attenuated IL-2 signaling and promote the differentiation of memory-phenotype cells. In summary, DCs are likely to contribute to the fate determination of CD8^+^ T cells by regulating IL-2 signaling, especially at the initial priming phase.

Long-term survival of memory CD8^+^ T cells is mediated by homeostatic proliferation (45). Our *in vitro* coculture analysis revealed that P14_moDC_ expressed high levels of CD122 and CD132, which are the subunits of IL-15 receptor, compared to P14_cDC_. As IL-15 plays an important role in maintaining memory T cells (46, 47), it might have been involved in the long-term survival of P14_moDC_ when P14_moDC_ were adoptively transferred to mice. Although IL-15Rα was not detected in P14_moDC_, other IL-15Rα-expressing cells could have delivered IL-15 signaling to P14_moDC_ via trans-presentation (48). Thus, we suggest that moDCs direct CD8^+^ T cells to express IL-15 receptors, enabling CD8^+^ T cells to survive at the memory phase.

The mechanisms for effector/memory differentiation of CD8^+^ T cells have been incompletely understood. Asymmetric cell division, which directs proximal and distal daughter cells to differentiate into SLECs and MPECs, respectively, has recently emerged as one of the mechanisms explaining how effector and memory progenies occur from naïve parental T cells (26, 49). A previous report suggested that strong TCR stimulation is required for the initiation of asymmetric cell division of CD8^+^ T cells. On the contrary, weak TCR stimulation preferentially leads to symmetric cell division of CD8^+^ T cells, resulting in the increased generation of MPECs (50). These results suggest that different TCR stimulation capacity of each DC subset could determine the fate of CD8^+^ T cells through differential regulation of the symmetry of cell division. In this regard, it would be interesting to compare the asymmetry in T cell division upon stimulation with different DC subsets.

We used *Ccr2*^−/−^ mice to define the roles of moDCs in CD8^+^ T cell differentiation during infection *in vivo*. The reduced memory CD8^+^ T cell responses in *Ccr2*^−/−^ mice correlated with our *in vitro* studies that demonstrated the specialized role of moDCs in the induction of memory precursor cells. We were unable to rule out the contribution of monocytes to memory CD8^+^ T cell differentiation because *Ccr2*^−/−^ mice exhibited a decreased frequency not only of moDCs but also of monocytes (Supplementary Figure 3B). However, monocytes themselves have been considered as less efficient APCs in CD8^+^ T cell stimulation (51). Monocytes can uptake foreign antigens, but they present antigens to T cells after subsequent differentiation into moDCs (52). Although a recent report have suggested that Ly6C^+^ monocytes can prime CD8^+^ T cells efficiently, a majority of Ly6C^+^CD11b^+^ cells in that report showed the feature of moDCs that express a certain level of CD11c (16). Thus, we suggest that reduction in moDCs is mainly responsible for defective memory CD8^+^ T cell formation in *Ccr2*^−/−^ mice.

It should be noted that the role of monocyte-derived cells in establishing defense mechanisms against pathogens is context-dependent. A previous report demonstrated that Tip-DCs, which share many phenotypic characteristics with moDCs in our experimental setting, mediate innate immune responses but are dispensable for T cell priming in Lm infection (13). We also showed that CD8^+^ T cells primed in *Ccr2*^−/−^ mice exhibited no defects in their cytokine production capacity and cytotoxicity during LCMV-Arm infection. However, we identified an unrecognized role of moDCs in triggering memory CD8^+^ T cell generation under cognate antigenic stimulation. Interestingly, during the late phase of LCMV chronic (CL-13) infection, monocytes have been shown to acquire a myeloid-derived suppressor cell (MDSC)-like feature that abrogates CD8^+^ T cell proliferation and drives T cell exhaustion (17). The underlying factors that educate monocytic cells to play opposing roles during acute and chronic infections remain to be elucidated.

Taken together, our study demonstrates a crucial role of moDCs in the generation of memory CD8^+^ T cells during acute antiviral immune responses. Our findings expand the understanding of the link between myelopoiesis and CD8^+^ T cell differentiation during acute viral infection and have implications for the development of novel vaccine strategies against infection.

## Data Availability

The raw data supporting the conclusions of this manuscript will be made available by the authors, without undue reservation, to any qualified researcher.

## Ethics Statement

All animal experiments were approved by and carried out in accordance with the approved guidelines of the Institutional Animal Care and Use Committee (IACUC) of Seoul National University.

## Author Contributions

K-SS and C-YK designed the study. K-SS, IJ, C-HK, BS, J-ML, JL, E-AB, and HS performed experiments. K-SS, B-SK, Y-JP, and C-YK analyzed and interpreted the data. YB and S-JH provided reagents. K-SS, I-KK, B-SK, and C-YK wrote the manuscript. C-YK supervised the study.

### Conflict of Interest Statement

The authors declare that the research was conducted in the absence of any commercial or financial relationships that could be construed as a potential conflict of interest.
