# Supplementary material for: Monocyte-Derived Dendritic Cells Dictate the Memory Differentiation of CD8+ T Cells During Acute Infection
Source: Front Immunol. 2019 Aug 16;10:1887. doi: 10.3389/fimmu.2019.01887 (PMC6706816; doi:10.3389/fimmu.2019.01887)
Supplement: Supplementary file 1 [file Data_Sheet_1.pdf]

Supplementary Material

1 Supplementary Figures

Supplementary Figure 1. Shin *et al.*

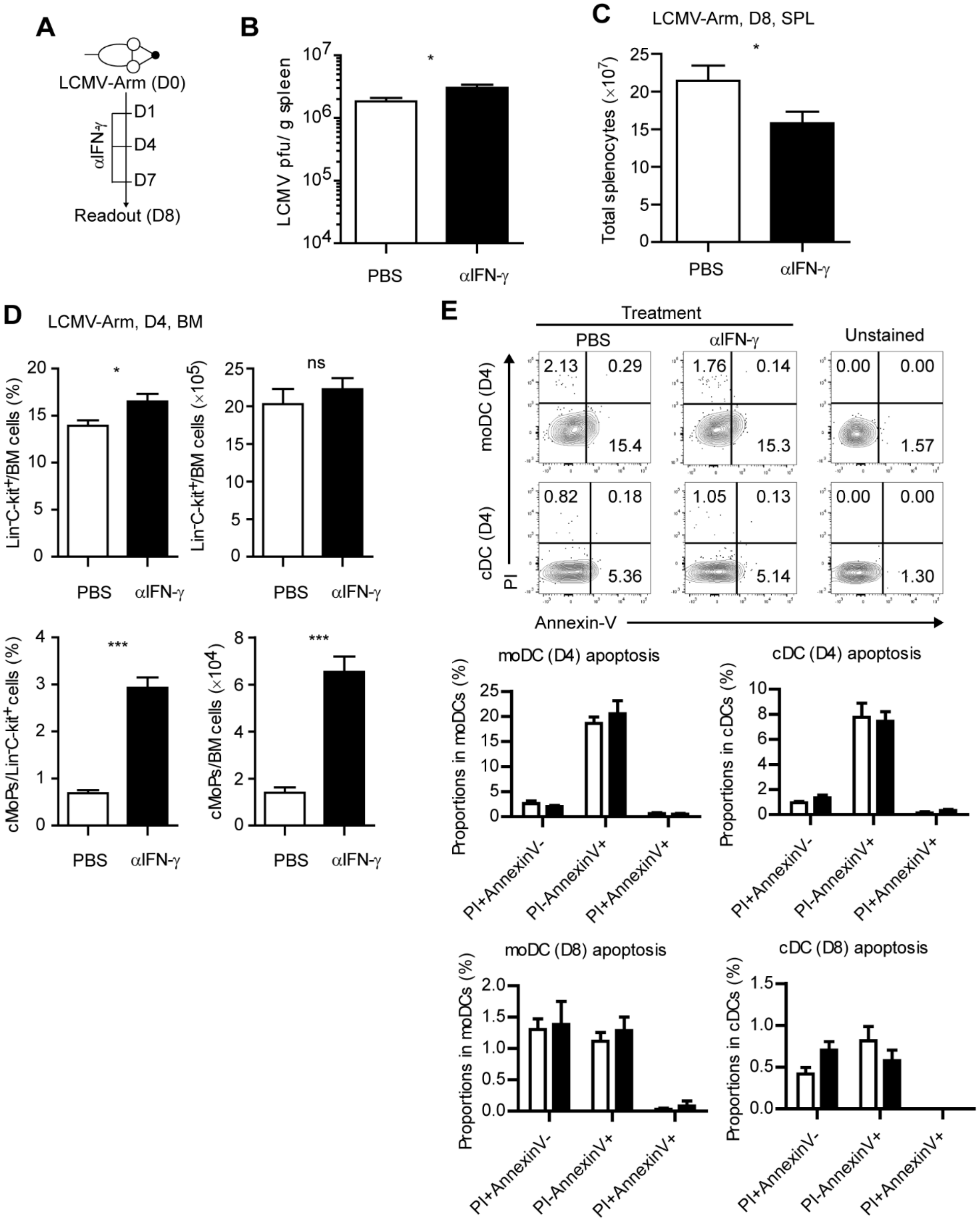

Supplementary Figure 1. IFN-γ neutralization-induced changes in LCMV-Arm-infected mice
(**A**) Experimental schedule. (**B**) Viral titers in the spleen of the mice that received IFN-γ–neutralizing Abs or not were calculated by the plaque assay at day 4 p.i.(**C**) Splenocyte numbers of the mice received IFN-γ–neutralizing Abs or not. (**D**) Cell numbers and frequencies of $Lin^{-}C\text{-}kit^{+}$ BM progenitor cells and cMoPs in the BM of mice. (**E**) Propidium Iodide (PI) and Annexin-V stainings of moDCs and cDCs from infected mice that received IFN-γ–neutralizing Abs or not. Representative flow cytometry plots (upper) and graph plots (lower). Numbers in the flow cytometry plots indicate the percentages within the gates. n=5 or 6 per group. Data are shown as the mean±SEM. *p<0.05; **p<0.01; ***p<0.001.

Supplementary Figure 2. Shin *et al*.

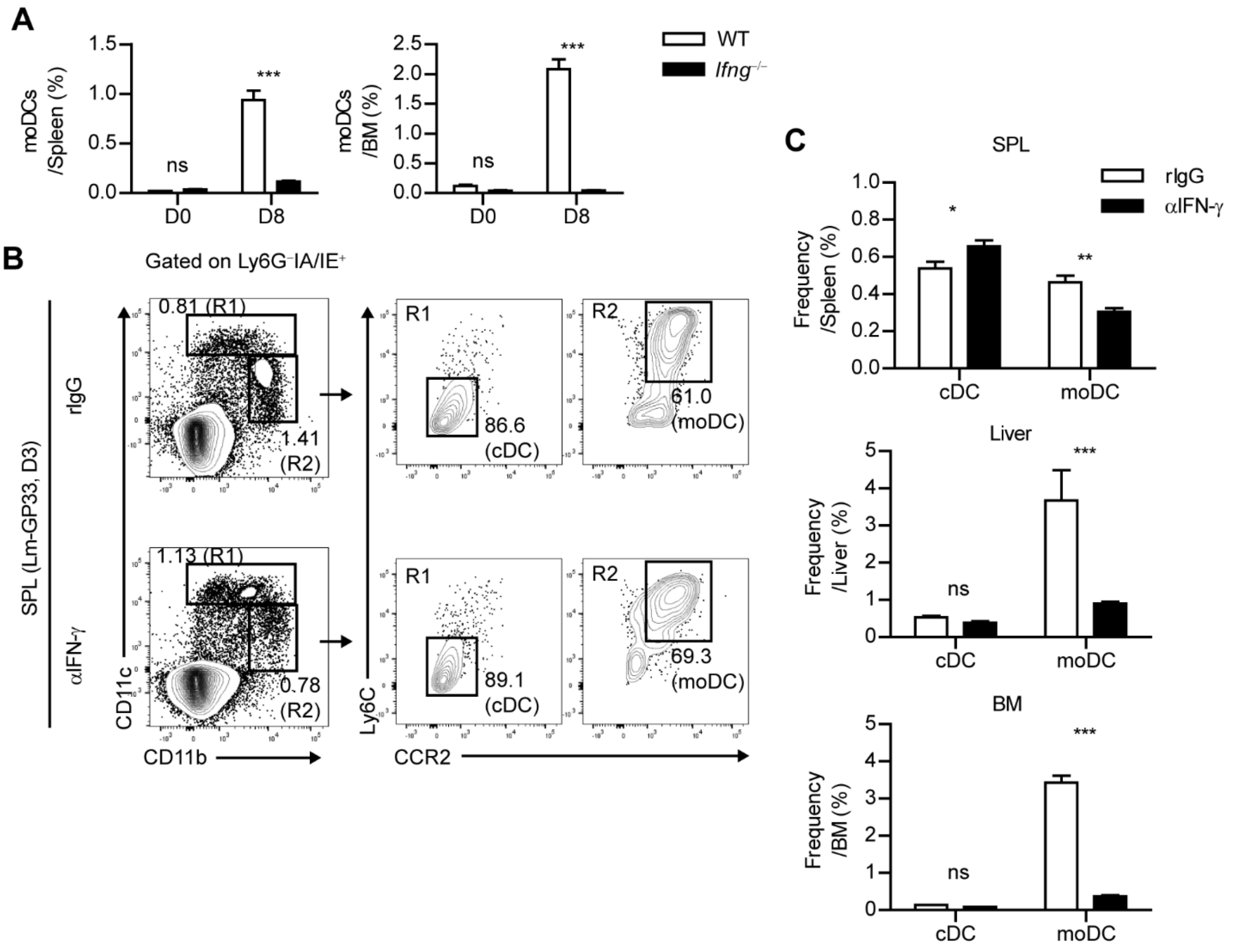

Supplementary Figure 2. IFN-γ-dependent expansion of moDCs during infection
(**A**) WT and *Ifng*$^{-/-}$ mice were infected with LCMV-Arm, and analyzed on day 8 p.i. Graphs of the frequencies moDCs in the spleen and BM are shown. n=3 per group. (**B and C**) Mice were infected by Lm-GP33 followed by the neutralization of IFN-γ with mAbs. The frequencies of cDCs and moDCs on day 3 p.i. are shown as flow cytometry plots (**B**) and graphs (**C**). Numbers in the flow cytometry plots indicate the percentage within each gate. n=5 per group. Data are representative of two independent experiments and are shown as the mean±SEM. *p<0.05;**p<0.01; ***p<0.001.

Supplementary Figure 3. Shin et al.

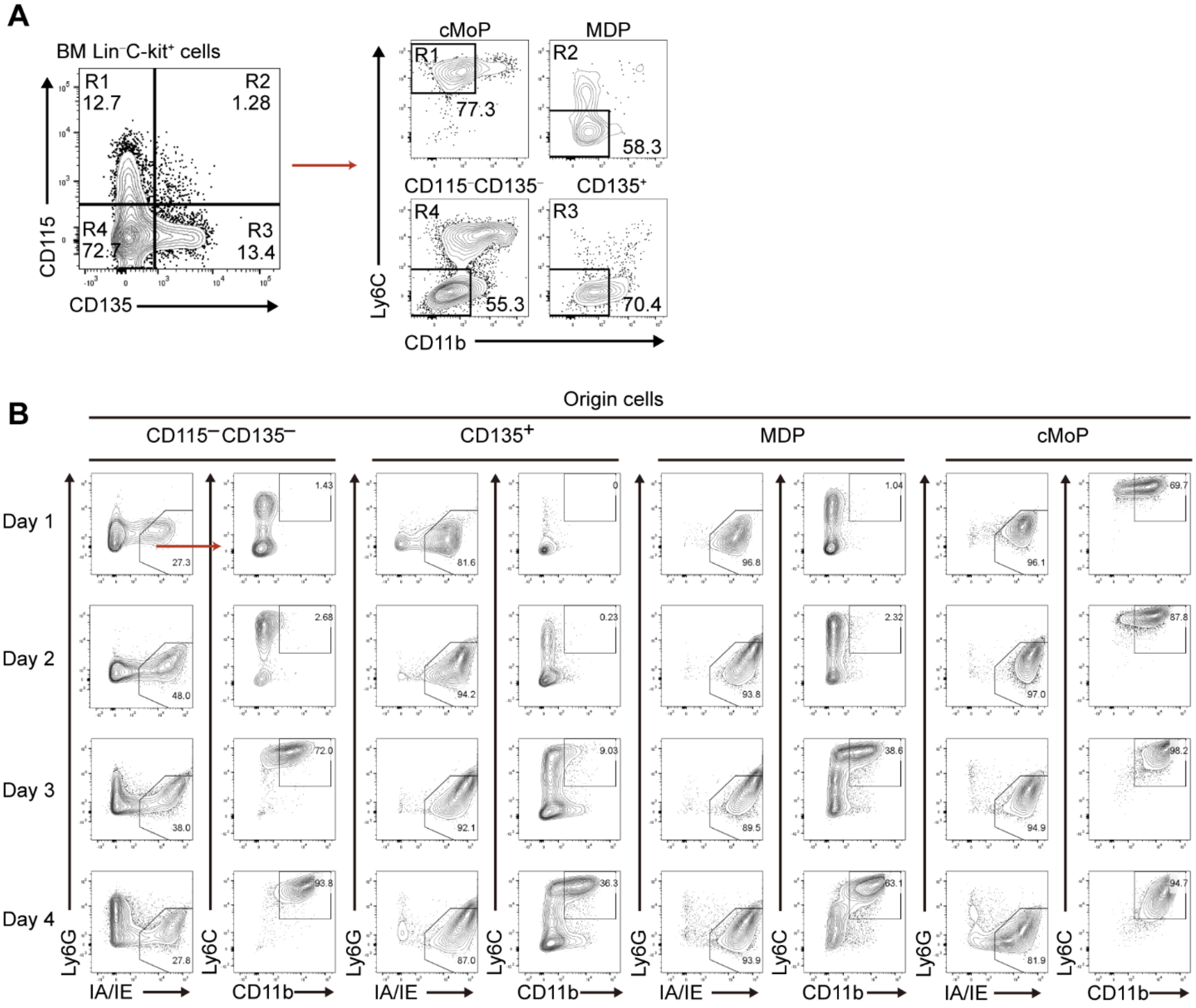

Supplementary Figure 3. Daily differentiation of the subdivided BMP subsets into moDCs under GM-CSF and IFN-γ stimulation

(**A**) Gating and sorting strategy for $CD115^{-}CD135^{-}$, $CD135^{+}$, MDP and cMoP in BMPs. Numbers in the plots indicate the percentages of the cells within the gates. (**B**) BMP subsets were sorted as in (A), and their differentiations into moDCs under GM-CSF and IFN-γ stimulation were analyzed daily. Data are shown as flow cytometry plots. Numbers in the plots indicate the percentages of the cells within each gate.

Supplementary Figure 4. Shin *et al.*

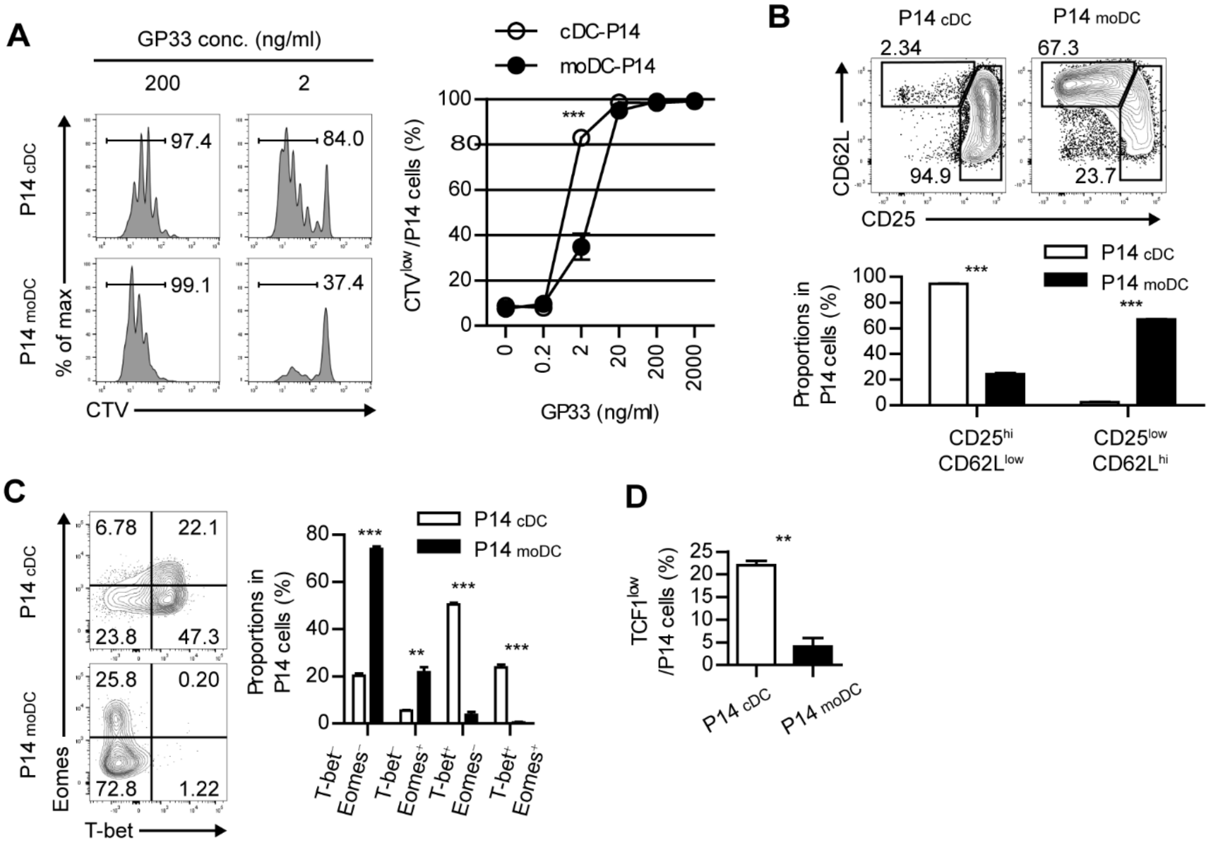

Supplementary Figure 4. Differentiation patterns of P14 cells primed by moDCs that were isolated from LCMV-Arm-infected mice at day 8 p.i.
moDCs and cDCs were isolated from LCMV-Arm-infected mice at day 8 p.i. and cocultured with P14 cells in the presence of $GP_{33\text{-}41}$ peptide for 3 days. (**A**) Dividing patterns of P14 cells primed by day 8 cDCs or moDCs are shown as histogram plots (left) and a graph plot (right). Numbers in the histogram plots indicate the percentages of divided cells in each condition. (**B**) Coexpressions of CD25 and CD62L on P14 cells primed by cDCs or moDCs are shown as flow cytometry plots (upper) and a graph plot (lower). (**C**) Coexpressions of T-bet and Eomes of P14 cells primed by day 8 cDCs or moDCs are shown as flow cytometry plots (left) and a graph plot (right). Numbers in the flow cytometry plots indicate the percentages of each quadrant. (**D**) TCF1 expression of P14 cells primed by day 8 cDCs or moDCs is shown as a graph plot of $TCF1^{low}$ cells. Data are representative of three independent experiments and are shown as the mean±SEM. $^{**}p<0.01$; $^{***}p<0.001$.

Supplementary Figure 5. Shin *et al*.

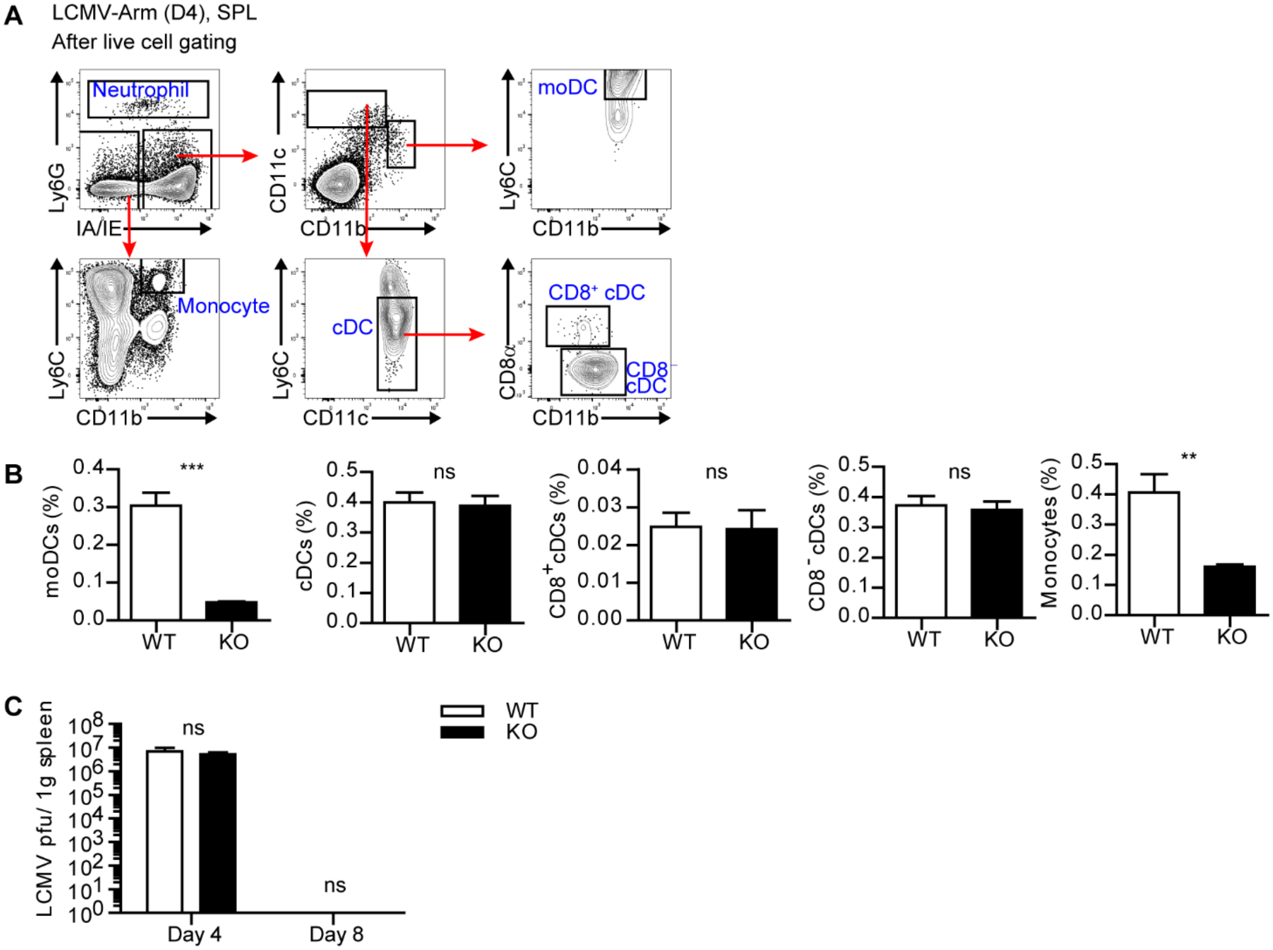

Supplementary Figure 5. CCR2-deficient mice exhibit reduced numbers of moDCs and monocytes during LCMV-Arm infection

(**A**) Gating strategy for moDCs, monocytes and cDCs in the splenocytes of LCMV-Arm-infected mice 4 days p.i. (**B**) The frequencies of moDCs, cDCs and monocytes of LCMV-Arm-infected WT and *Ccr2*$^{-/-}$ mice are shown as graphs. (**C**) Viral titers in the spleen of WT and *Ccr2*$^{-/-}$ mice on day 4 and day 8 p.i. were calculated by the plaque assay. Data are representative of two independent experiments and are shown as the mean±SEM. n=5 per group. **p<0.01; ***p<0.001.

Supplementary Figure 6. Shin *et al*.

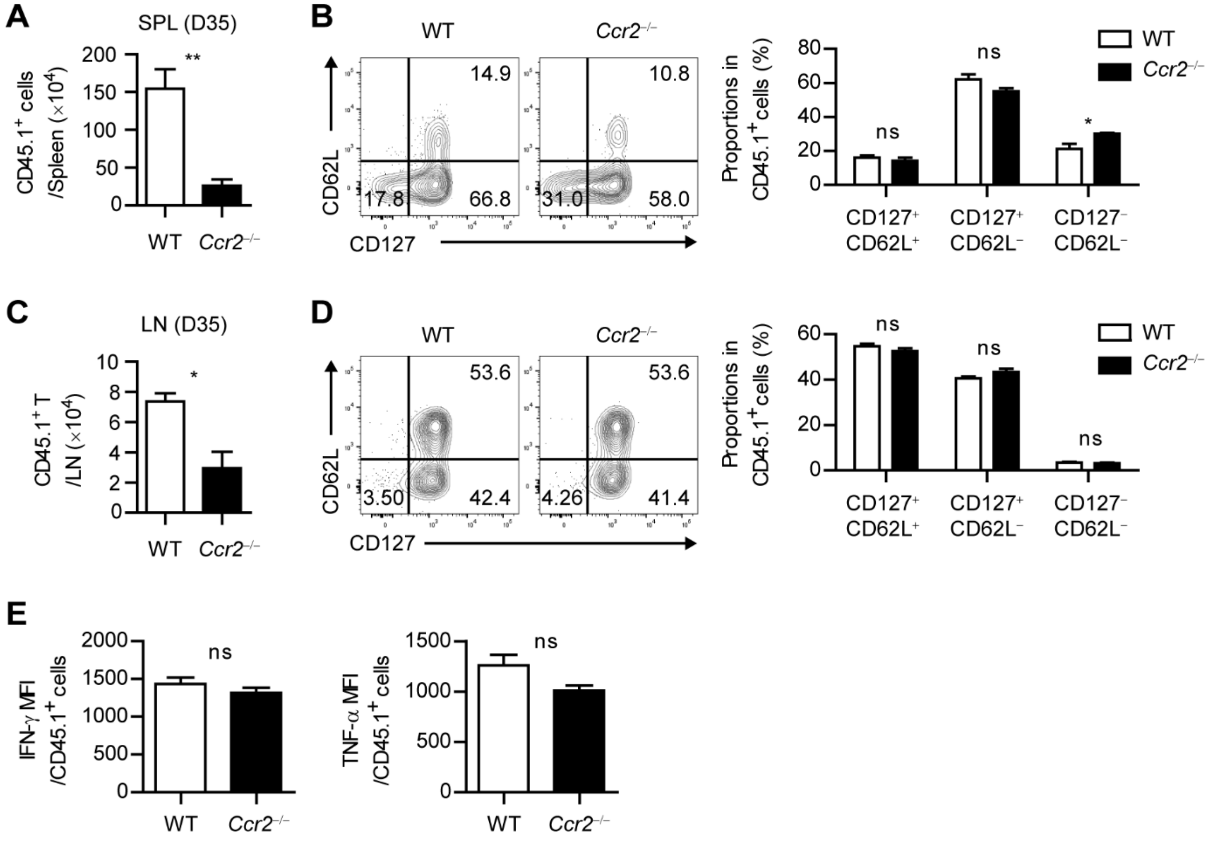

Supplementary Figure 6. CCR2-deficient mice fail to generate proper CD8$^+$ T memory without affecting their quality

WT and *Ccr2*$^{-/-}$ mice containing CD45.1$^+$ P14 cells were infected with LCMV-Arm and donor P14 cells were analyzed on day 35 p.i. (**A-D**) The numbers of CD45.1$^+$ P14 cells was determined in the spleen (**A**) and LN (**C**) and the coexpression of CD62L and CD127 of CD45.1$^+$ P14 cells was analyzed in the spleen (**B**) and LN (**D**) of WT and *Ccr2*$^{-/-}$ mice. Numbers in the flow cytometry plots indicate the percentage of each quadrant. (**E**) Secretion levels of IFN-γ and TNF-α in CD45.1$^+$ P14 cells in the spleen of WT and *Ccr2*$^{-/-}$ mice are shown as graphs. Data are representative of two independent experiments and are shown as the mean±SEM. n=3-4 per group. *p<0.05;**p<0.01.

Supplementary Figure 7. Shin *et al*.

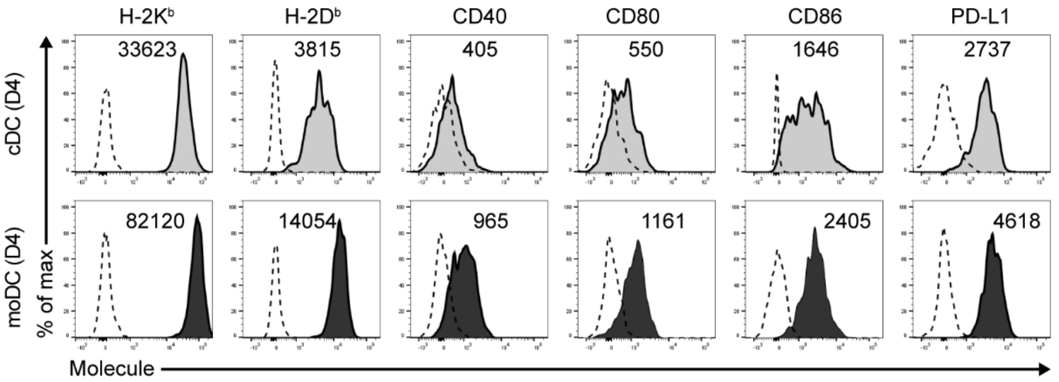

Supplementary Figure 7. Expression levels of surface molecules involved in T cell signaling of cDCs and moDCs

The expression levels of MHCI (H-2K^{b} and H-2D^{b}), costimulatory (CD40, CD80 and CD86) and coinhibitory (PD-L1) molecules of cDCs and moDCs from LCMV-Arm infected mice at day 4 p.i. were measured by flow cytometry and are shown as histograms. Numbers in the histograms indicate the MFI values of each molecule. Dashed histograms indicate isotype Ab staining.

Supplementary Figure 8. Shin *et al*.

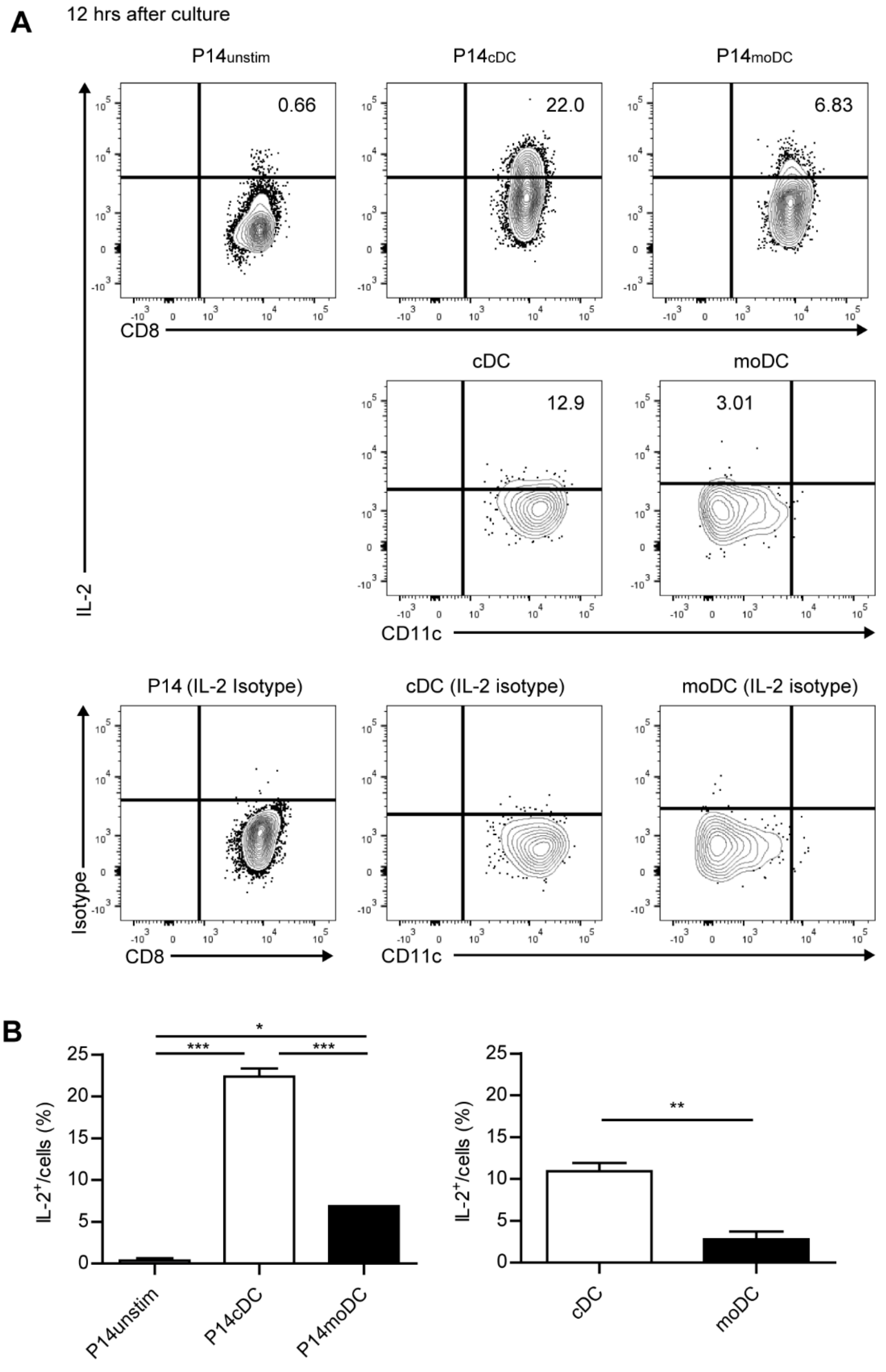

Supplementary Figure 8. IL-2 secretion levels of T cells and DCs in the early time point of cocultures

moDCs and cDCs isolated from LCMV-Arm-infected mice were cocultured with P14 cells in the presence of $GP_{33\text{-}41}$ peptide, and IL-2 secretion levels of P14 cells and of each DC subset were determined by intracellular cytokine staining at 12 hours after culture. GolgiPlug were added to

culture 4 hours prior to analysis. (**A**) Representative flow cytometry plots. Numbers in the plots indicate the IL-2^+ percentages of indicated subsets. (**B**) Graph plots of IL-2^+ cells within each T cell and DC subset. Data are shown as the mean±SEM. *p<0.05;**p<0.01;***p<0.001.
